# The necessity of incorporating non-genetic risk factors into polygenic risk score models

**DOI:** 10.1038/s41598-023-27637-w

**Published:** 2023-02-20

**Authors:** Sipko van Dam, Pytrik Folkertsma, Jose Castela Forte, Dylan H. de Vries, Camila Herrera Cunillera, Rahul Gannamani, Bruce H. R. Wolffenbuttel

**Affiliations:** 1 Department of Endocrinology, University of Groningen, University Medical Center Groningen, P.O. Box 30001, 9700 RB Groningen, The Netherlands; 2 Ancora Health B.V., Herestraat 106, 9711 LM Groningen, The Netherlands; 3 Department of Clinical Pharmacy and Pharmacology, University of Groningen, University Medical Center Groningen, P.O. Box 30001, 9700 RB Groningen, The Netherlands; 4 Department of Neurology, University of Groningen, University Medical Center Groningen, P.O. Box 31000, 9700 RB Groningen, The Netherlands

**Keywords:** Diabetes, Population genetics, Genetics, Risk factors, Diseases, Cancer, Cardiovascular diseases, Endocrine system and metabolic diseases

## Abstract

The growing public interest in genetic risk scores for various health conditions can be harnessed to inspire preventive health action. However, current commercially available genetic risk scores can be deceiving as they do not consider other, easily attainable risk factors, such as sex, BMI, age, smoking habits, parental disease status and physical activity. Recent scientific literature shows that adding these factors can improve PGS based predictions significantly. However, implementation of existing PGS based models that also consider these factors requires reference data based on a specific genotyping chip, which is not always available. In this paper, we offer a method naïve to the genotyping chip used. We train these models using the UK Biobank data and test these externally in the Lifelines cohort. We show improved performance at identifying the 10% most at-risk individuals for type 2 diabetes (T2D) and coronary artery disease (CAD) by including common risk factors. Incidence in the highest risk group increases from 3.0- and 4.0-fold to 5.8 for T2D, when comparing the genetics-based model, common risk factor-based model and combined model, respectively. Similarly, we observe an increase from 2.4- and 3.0-fold to 4.7-fold risk for CAD. As such, we conclude that it is paramount that these additional variables are considered when reporting risk, unlike current practice with current available genetic tests.

## Introduction

### Risk perception to stimulate preventive health action

Chronic disease is an ever-growing problem in western society, with 32–58 percent of all Europeans age 50 and over suffering from multiple age-related non-transmissible chronic diseases^1^. These chronic conditions can in part be prevented by following simple health guidelines such as regular physical exercise, having a healthy diet, and not smoking^2–4^. Yet, the adherence to this advice is limited. Among other reasons, this can be explained by the low perceived risk for each of these chronic conditions separately^5^, which can influence health behaviors^6–8^ and, in turn, lower risk for chronic disease^9^.

There is a growing interest in genetics-based risk assessment, as evident by the over 27 million genetic tests sold worldwide^10^. This growing interest, combined with the predictive power of polygenic scores (PGS), can and is harnessed to promote disease prevention^8,11,12^.

PGS are risk scores, computed based on genetic profiles and have proven effective at identifying individuals (10% individuals at highest risk) with a 2.5 and 2.9 odds ratio for developing type-2-diabetes (T2D) and coronary artery disease (CAD), respectively, when compared to the rest of the population. These risk assessments are based on relatively cheap genotyping chip assessments (as opposed to whole genome sequencing (WGS) required for monogenic analyses), well suited for PGS calculations^13–15^. Indeed, these PGS are now being implemented in commercially available tests^16,17^ and made available to the public.

### Genetic health risk limitations

Although PGS have proven able to identify individuals at high risk based on genotyping chip data, the usefulness of this newer approach to risk stratification remains a topic of debate^18,19^. One commonly raised concern is that the variance explained for the predicted outcomes is often low. Typically, these vary between 1 to 5% for phenotypes such as diabetes and CAD^19,20^. Other, non-genetic risk factors, such as age, sex, smoking status, parental disease status, physical activity and body mass index (BMI), which already form part of most clinical risk prediction models, have proven more effective at identifying individuals at high risk^21–25^. Combining both genetic and non-genetic factors leads to improved risk prediction, vastly increasing the discriminative power^26–28^. Visa versa, the added value of PGS was only limited to these existing models. As a result of the established limited added predictive power resulting from adding PGS to existing risk models^27,29^, to date they are rarely used in the clinical setting. On the other hand, public interest in genetic risk has increased as evidenced by the billion-dollar companies selling PGS commercially, resulting in more disease risk awareness^8^.

Unfortunately, to date, commercially available genetic risk assessments do not leverage information of additional risk factors to improve the predictions. One limitation of the best performing risk models, is that they usually also require biomarker measurements, which are a great barrier to implementation. Fortunately, it was previously shown PGS models can also be vastly improved when only variables that can be easily attained, e.g. through a simple questionnaire, even with a single variable such as BMI^30^. Since risk predictions can affect health behavior and decision making of individuals^31^, models that include easily acquirable variables in addition to PGS should be deployed by these commercial parties.

While some models, solely based on variables attainable through genotyping and questionnaire data, exist^32^, two limitations, still remain. The first limitation is that much of the previous work was solely conducted on a single dataset, often the UK Biobank data, and an external validation is important for a variety of reasons^33,34^. Second, PGS based risk calculation methods to date require a large reference cohort to translate arbitrary PGS scores into disease risk estimates, by means of calibration. In order conduct this translation this reference dataset needs to use genotyping data based on the same genotyping chip, which is not always available. But even when they do have access to a large biobank, this limits the possible use of genotyping chips to only those readily used by large biobanks and additionally causes trouble when one would like to compare genotyping chips to multiple biobanks. The latter will become more and more desirable, as more biobanks for different ethnicities become available, since PGS based risk assessments should be based on a reference cohort of the same ethnicity. To allow feasible implementation of PGS based risk assessment in practice in any multi-ethnic population it is therefore important to use a method that allows for this. To this end we constructed a method that circumvents this problem. To validate this indeed is effective and that models including additional risk factors, limiting ourselves to those that allow for feasible implementation in practice, constructed in the UK Biobank (UKB)^35^ are also usable outside this context, we validate our results externally using the Lifelines data^36^.

## Results

### Study outline

We have built predictive models using Cox regression^37^, including and excluding a number of easily attainable variables, using the UKB data (with tenfold cross validation). We built models separately for prediction of T2D and CAD. All models were trained using the UKB and tested in the Lifelines data (for details see Supplementary materials). The UKB and Lifelines are two large databases for which numerous statistics are available, among which the input variables required for our models for a large number of individuals: genotyping chip data, BMI, genetic sex, smoking status, quantification of physical activity, parental disease status (Table 1). All reported statistics refer to the results for the Lifelines data used for testing the performance of the models trained in the UKB, unless specified otherwise.

**Table 1:** Statistics of included participants. Data are presented as mean (SD) or n (%). For a histogram of the age distributions, we refer to Supplementary Fig. 1.

	UKB	Lifelines
Number of included individuals	406,159	36,130
Number of males	186,493 (45.9%)	15,004 (41.5%)
Number of females	219,666 (53.8%)	21,126 (58.5%)
Age range (years)	38–75	5–91
Body mass index (BMI), kg/m^2^	27.4 (SD:4.8)	25.0 (SD:4.6)
Number of individuals currently smoking	41,105 (10.12%)	6,203 (17.2%)
Number of individuals smoking in the past	184,388 (45.4%)	11,001 (30.4%)
Average days/week with vigorous activity	1.7	2.2
Average days/week with moderate activity	3.4	4.2
T2D prevalence at first assessment	20,118 (5.0%)	547 (1.5%)
T2D incidence after first assessment	8363 (2.1%)	270 (0.75%)
CAD prevalence at first assessment	13,648 (3.4%)	516 (1.43%)
CAD incidence after first assessment	9027 (2.2%)	255 (0.71%)

**Figure 1:**
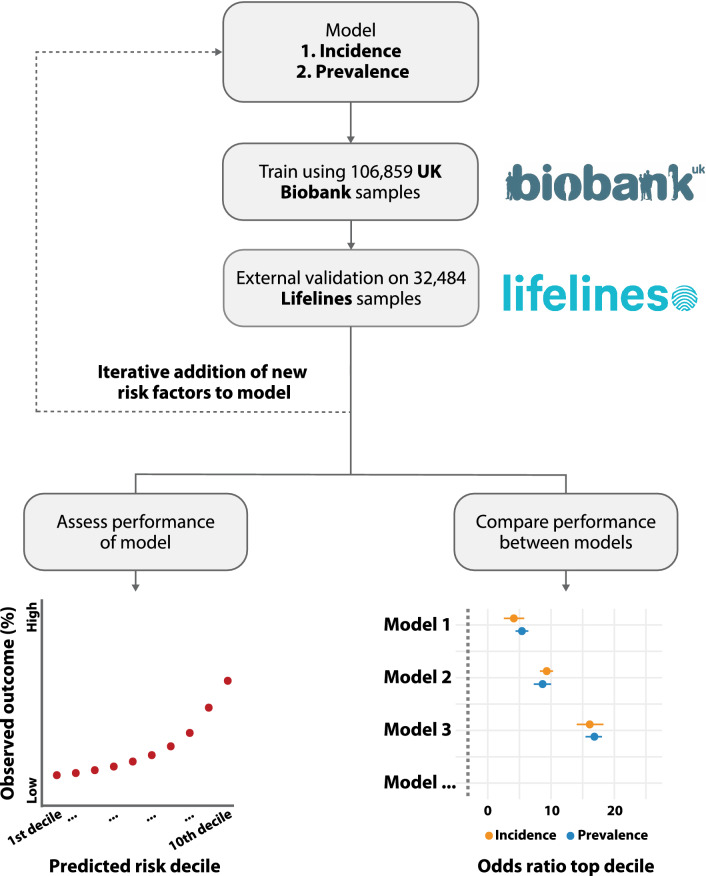
Training and validation setup study (concept). Models were trained based on a subset of UK Biobank individuals and validated in both the remainder of the UK Biobank individuals and the Lifelines cohort.

All analyses were conducted twice, once to model incidence and once to model prevalence (Fig. 1). Predicting prevalence for T2D is less appropriate for the purpose of prevention as T2D also impacts the risk factors. E.g., individuals with T2D are more likely to become obese, while obesity also increases risk for T2D^38^. Additionally, PGS perform different at predicting prevalence versus incidence^39^. We primarily focus our analyses on predicting incidence rather than prevalence. To model prevalence, we used the entire dataset. To study incidence, we exclude all individuals that had already attained the respective outcome on their first visit.

**Figure 2:**
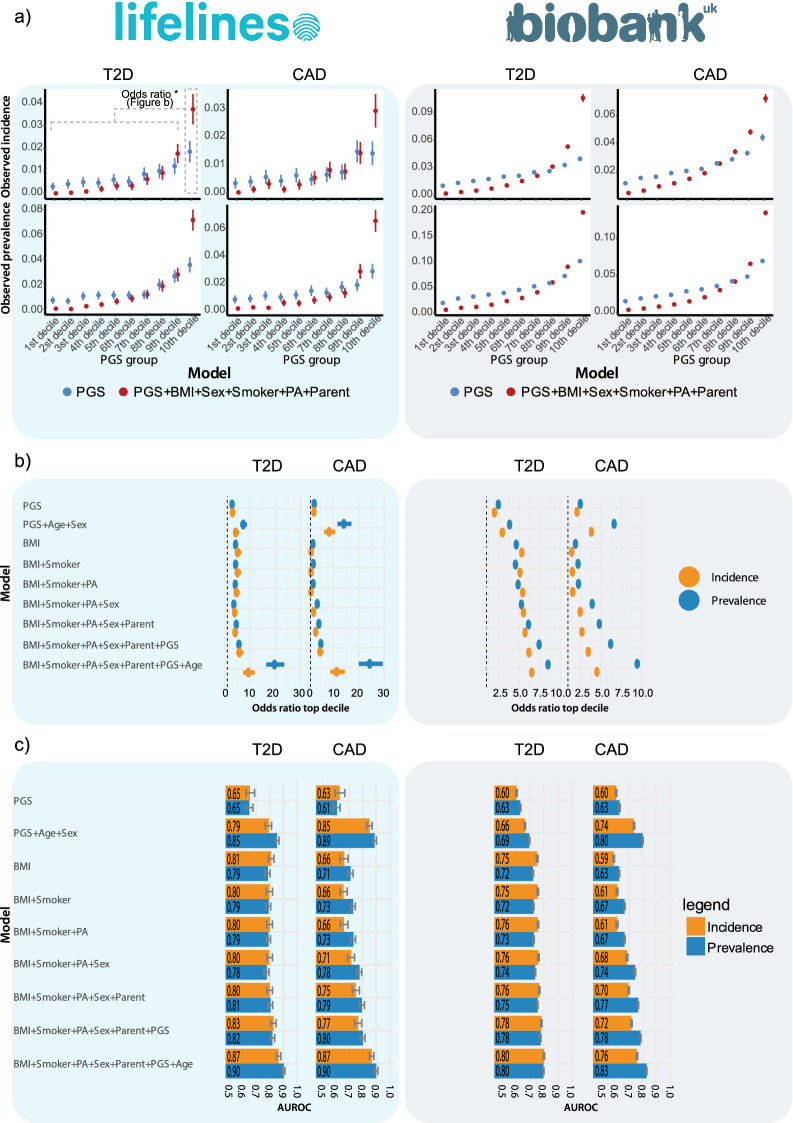
Comparison of models including only PGS or additional variables that can be attained through a questionnaire. Risk predicted with questionnaire-based variables performs similar or better at identifying individuals at high risk (10th decile), compared to PGS. Adding PGS to a questionnaire-based model can, however, further improve the identification of high-risk individuals, but requires a large dataset to be detectable due to its limited effect. For a comparison of risk in different risk strata at different ages, we refer to Supplementary Fig. 2. (**A**) Absolute incidence and prevalence per decile based on PGS alone or combine with additional variables. Performance improves if additional variables are added beyond PGS alone. Risk is increasing exponentially in higher risk categories. (**B**) Odds and incidence ratios of individuals in the top decile according to different models. Model including questionnaire-based risk variables performs significantly better at identifying individuals that will get the respective outcome than a model based on PGS alone. (**C**) C-indexes of the different models. Added value of PGS on top of variables that can be derived from a questionnaire is limited. *PGS* Polygenic risk score, *BMI* Body mass index, *C-index* Harrell’s C-index, *PA* Physical activity (based on number of days moderate and days of vigorous activity), *Parent* Parental T2D status, Variables not included as predictors in the model were included as covariates. Additionally, in the UK Biobank, data the first 4 PCs and genotyping batch were included as covariate. Bars indicate 95% confidence interval. For numerical representation we refer to the Supplementary Table 1.

We present our results as incidence odds ratios of individuals in the highest risk decile compared to the remainder of the population (Fig. 2b), to allow for comparison to previous works and easy interpretability. Furthermore, individuals at highest risk stand to gain most from intervention, which makes identifying this group highly relevant. Additionally, we report the Harrel’s C-index^40^ for all different models (Fig. 2c, Supplementary Table 1).

### PGS based predictions

**Figure 3:**
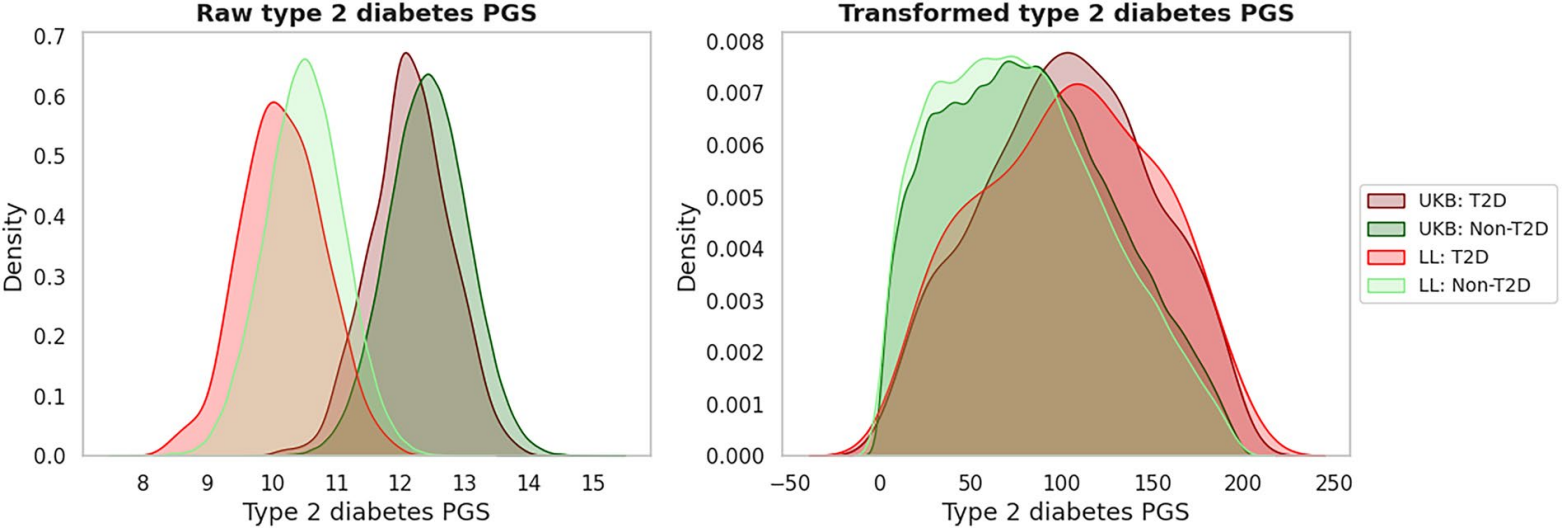
Absolute PGS scores based on different genotyping chips cannot be compared directly. Left: Raw summed PGS scores; Risk scores calculated based on different genotypes are largely different, causing a bias when a risk assessment based one type of chip is compared to a reference cohort based on a different chip. Note that scores for individuals with T2D are lower than those without T2D as the calculated PGS scores capture the protection against T2D (can be multiplied by − 1 to indicate sensitivity for T2D rather than protection). Right: Raw PGS scores were compared to raw PGS scores of the individuals of the 1000 g cohort to translate them into percentiles. This was done for both PGS scores (based on GWAS summary statistic file including variants with p-value threshold 0.01 and 10^–6^). Then the percentiles where summed and the densities plotted. Raw PGS scores UKB diabetes versus Lifelines diabetes are statistically significantly different (p-value < 10^–323^, Mann–Whitney U test test), but summed PGS for UKB diabetes versus Lifelines diabetes are not (p-value: 0.51, two sided Mann–Whitney U test t-test). *PGS* Polygenic Risk Score.

First, we show projecting PGS scores, based on a genotyping chip, against a reference cohort, based on WGS data, allows chips with different markers to be put onto the same distribution. This effectively normalizes the PGS scores of the different chips, allowing them to be compared against each other. Prior to this normalization the PGS values a genotyping chip from the Lifelines data cannot be compared to that of the UKB, as the resulting bias would cause all Lifelines individuals to appear at high risk for T2D. On the other hand, after this normalization the distributions are very comparable allowing a genotyping chip from the Lifelines data (or any other dataset) to be compared against that of the UKB, or visa versa (Fig. 3). This method also can also be implemented for single genotyping chips that are not part of a bigger cohort.

Next, we reproduced earlier reports showing that PGS scores calculated through this normalization method can be used to identify high-risk individuals in Lifelines^13^. We trained and validated models in the UKB and then validated them also externally in the Lifelines cohort. In Lifelines, we observe that the prevalence odds ratio for those in the top decile for T2D and CAD are 2.9 (95-CI 2.3–3.6) and 2.5 (95-CI 2.0–3.1) with a C-index of 0.90 (95-CI 0.89–0.91) and 0.90 (95-CI 0.89–0.91) after correcting for age, sex, genotyping batch number, smoking status, physical activity, parental disease status and the first 4 principal components, respectively (Fig. 2c).

### Questionnaire-based risk factors improve incidence predictions based on PGS

Next, we investigate how much predictive power these PGS models would gain by including easily and freely attainable regular risk factors into these PGS-based models. We built a number of models to assess the added value of each of those variables, by integrating individual factors into the PGS-based model and by integrating PGS into the non-genetic factor model.

We are interested in identifying individuals at high risk of obtaining T2D or CAD in the future, aiming to act preventively in high-risk individuals. To create models that are suited for identification of individuals, of a certain age, at risk of obtaining either T2D or CAD (rather than already having it), we trained the model on incidence (as opposed to prevalence)^26^. Prior to our analyses, we have thus removed individuals that have the outcome on their initial measurement from the data. For comparison, we have also created models that predict prevalence rather than incidence (Fig. 2).

For a T2D prediction model based on PGS, we observe that individuals in the highest risk decile have a 3.0 (95-CI 2.3–4.1) fold higher incidence, which increases to 5.8 (95-CI 4.5–7.4) when BMI, physical activity, sex, parental disease and smoking status are included in the model (Likelihood ratio Chi-square test p-value: 2.1 × 10^–20^).

In addition to the prior model, we constructed a model that includes age as an additional risk factor. We built this model separately as we deemed it of less value to compare individuals at different ages when aiming to identify individuals that would benefit most from preventive action. When age is also added to the model the incidence odds ratio in the top decile increases to 9.3 (95-CI 7.3–11.8, Likelihood ratio Chi-square test p-value: 3.0 × 10^–26^).

Similar to T2D, for CAD, Lifelines individuals in the highest risk decile have a 2.4-fold (95-CI 1.7–3.3) increased risk for CAD when modelling incidence based on PGS compared to 4.7 (95-CI 3.7–6.1) when BMI, physical activity, sex, parental disease and smoking status are also included in the model (Likelihood ratio Chi-square test p-value = 4.0 × 10^–08^).

When age is also included in the model the incidence odds ratio in Lifelines increases to 11.3 (95-CI 8.8–14.5, likelihood ratio Chi-square test p-value = 3.6 × 10^–33^). The effect of age is larger than in the UKB where the incidence odds ratios in the highest decile are 4.5 (95-CI 4.3–4.7, Fisher exact test p-value = 4.0 × 10^–39^). This is likely due to the much larger age range of the participants in the Lifelines database with the rarity of CAD at younger ages being much lower (5–91 year in Lifelines and 38–75 year in the UKB; for the age distribution we refer to Supplementary Fig. 1).

We conclude that there is a clear benefit of adding risk factors that can be obtained through a simple questionnaire to PGS-based risk assessments.

### Limited added value of PGS on top of questionnaire-based risk factors for prediction of incidence

In the previous section, we investigated the added benefit of adding questionnaire-based risk factors to PGS. Here, we investigate to what extent PGS add value to a model based on solely those non-genetic risk factors that can be attained through a questionnaire, to predict incidence. This will allow an assessment of the added value for the added cost and effort of running a genotyping chip.

For a T2D prediction model based on BMI, physical activity, sex, parental disease and smoking status we observe that, compared to the remainder of the population, individuals in the highest risk decile have a 4.0 (95-CI 3.1–5.2) fold higher incidence. When PGS are added to the model this increases to 5.8 (95-CI 4.5–7.4) fold (Likelihood ratio Chi-square test p-value = 2.2 × 10^–5^).

Similar to T2D, we modelled incidence for CAD based on BMI, physical activity, sex, parental disease and smoking status. In Lifelines, individuals in the highest risk decile have a 3.0 (95-CI 2.3–4.0) fold higher incidence compared to 4.7 (95-CI 3.7–6.1) when PGS are included in the model (Likelihood ratio Chi-square test p-value = 0.29). While the observed difference in the number of individuals in the highest risk decile is not significant, the addition of the PGS term to the model across the entire spectrum is (Likelihood ratio Chi-square test p-value = 3.7 × 10^–10^). This shows that PGS, to some extent, are exerting their risk effects through mechanisms that are not captured by these non-genetic risk factors.

Overall, it is clear that there is some, but limited, added value of PGS on top of questionnaire-based risk factors for predicting T2D and CAD incidence compared to when only free to attain risk factors are used. However, PGS are costly, logistically complex and is time consuming compared to the questionnaire which is cheap, fast and easy.

### PGS and non-genetic risk factors identify different aspects of disease risk

Previously, it was questioned whether PGS predict the same aspects of disease risk as these and other common, non-genetic risk factors^18^ and if PGS would thus be no more than a complex approach to achieve the same result. The fact that the PGS term is statistically significant in a model that contains also the other risk factor terms suggests that PGS capture some aspect of risk that is not already captured by non-genetic risk factors in this model. However, since the statistical significance of the term in the model can be difficult to interpret, we investigated whether individuals predicted to have a high incidence for T2D based on PGS alone are also identified through a model based on sex, smoking status and parental disease status. We investigated how the predictions from PGS compare to predictions based on BMI, sex and smoking, on an individual level.

**Figure 4:**
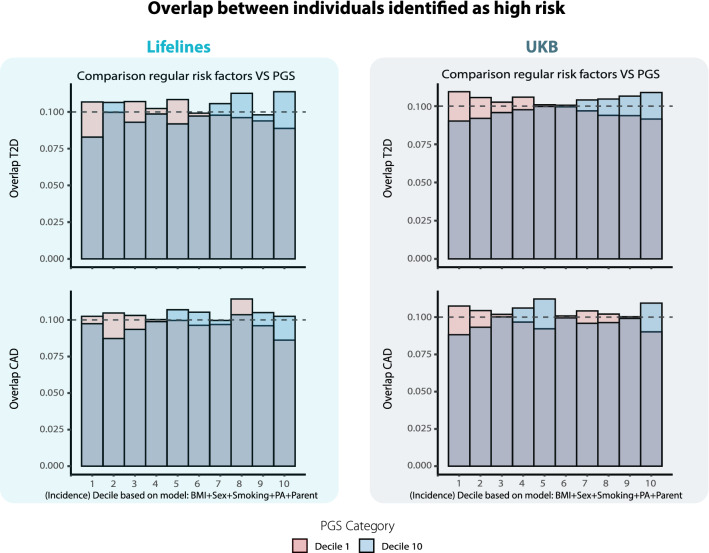
Comparison risk predictions based on a model based on PGS and a model based on sex, BMI, physical activity, parental T2D disease and smoking status. Each bar represents the percentage overlap of the individuals identified at high risk (10th) or low risk (1st decile) based on PGS compared to individuals identified at high risk according to questionnaire-based risk factors. Questionnaire-based risk factors identify other individuals at high risk than PGS. Dashed line indicates the overlap expected by random chance.

We found the correlation between the predictions of the model predicting risk based on a questionnaire data and a model predicting risk based on genetics is marginal (Lifelines: T2D: r = 0.03, p-value: 3.9 × 10^–07^; CAD: r = 0.02, p-value: 3.6 × 10^–4^, UKB: T2D: r = 0.03, p-value: 2.4 × 10^–93^; CAD: r = 0.02, p-value: 4.2 × 10^–33^). Over 60% of individuals ranked differing at least 3 deciles apart according to the two different models. Furthermore, approximately 7.5% of the individuals in the highest category based on the PGS based model (decile 1) were classed in the lowest risk category by non-genetic model (decile 10) (Fig. 4). Similar results are observed when prevalence, rather than incidence, is interrogated (Supplementary Fig. 3).

From our findings, we can conclude that risk predictions based on genetic risk scores are largely dissimilar to those derived from a list of known, questionnaire-based risk factors. While both predictions appear to allow identification of individuals at higher risk, they do largely disagree on whom those individuals are.

### Polygenic risk can be largely mitigated by controlling BMI for T2D and CAD

The fact that risk estimated based on questionnaire-based risk factors and risk based on genetics do not strongly overlap, suggests that non-genetic risk factors can be modified to mitigate the potential risk calculated based on genetics. To investigate whether individuals at high risk based on PGS can mitigate their genetic predisposition for T2D by adopting a healthier lifestyle, we investigated the effect of BMI in individuals in different genetic risk categories. We limited the analysis to BMI as, on the one hand, it is a known causal risk factor and showed largest impact in our analyses; and, on the other, weight reduction is a feasible lifestyle intervention which could be advised to mitigate genetic predisposition. Furthermore, limiting this analysis to the single most impactful variable allows for easy interpretation of the result.

**Figure 5:**
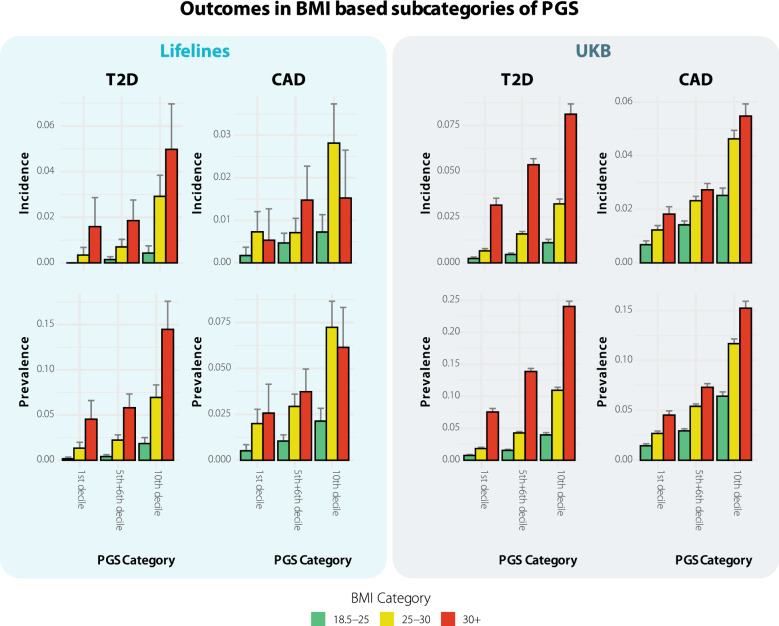
Comparison of diabetes incidence and prevalence in individuals with different genetic risk profiles and different BMI categories. Individuals with a high genetic risk profile benefit more from having a lower BMI in terms of absolute risk reduction, under the assumption that BMI is causal. Bars indicate 95% confidence interval.

We compared the effect of having a higher BMI in the different categories of genetic risk, in terms of both relative and absolute risk (Fig. 5). The T2D incidence in the low genetic risk category in those with a BMI above 30 was 1.6% and higher compared to the incidence of 0% among individuals with a BMI between 18.5 and 25 (Fisher exact test p-value: 1.6 × 10^–5^). In individuals at high genetic risk for T2D, the incidence of those with a BMI above 30 was 5.0% being higher than in those with a BMI between 18.5 and 25 which had an incidence of 0.4% (Fisher exact test p-value: 7.3 × 10^–11^). This indicates that the absolute difference in the high-risk group is threefold higher in the high genetic risk group compared to the low genetic risk group being only 1.6% in the prior compared to 4.6% in the latter group. A similar pattern is observed in the UK Biobank (Fig. 5). This suggests that those at high genetic risk for T2D benefit more from controlling their weight, than those having a low genetic risk.

For CAD we fail to observe this same phenomenon for incidence in Lifelines, but do observe this in case in the UKB (Fig. 5). The incidence in the low genetic risk group is 0.7% in the normal BMI (18.5–25) group and 1.8% in the high BMI (30+) group (Fisher exact test p-value: 2.1 × 10^–14^). The incidence in the high genetic risk group is 2.5% in the normal BMI group and 5.5% in the high BMI group (Fisher exact test p-value: 6.9 × 10^–28^). The absolute difference in the high genetic risk group is thus 3% compared to only 1.1% in the low genetic risk group. We ascribe our failure to observe this difference for incidence to the low incidence numbers in Lifelines. Taken together, this supports the notion that those at high genetic risk for CAD also benefit more from weight control than those in the low genetic risk group, in terms of absolute risk reduction.

### No significant interaction effects between PGS and other risk factors

In addition to the additive models, we have also created models including a multiplicative interaction term between BMI and PGS, but this term does not significantly contribute to the prediction of either T2D or CAD (Wald test p-value = 0.08). This is the case for both predicting prevalence and in case of predicting incidence. We do note that, although we do not observe these interactions to be significant, they may still exist but require larger sample sizes to detect, as large sample sizes are a known requirement for detecting interaction effects^41^.

## Discussion

We developed a method that allows different genotyping chips to be compared against different reference cohorts, without the need for having the same or any overlapping markers on the genotyping chip. This also allows assessment of risk from single genotyping chips when no reference cohort is available based on the same genotyping chip. We showed how this method can be combined with risk factors that are simple to acquire to predict risk much better at virtually no added cost or effort. This can help identify and motivate individuals that should be prioritized for preventive health measures.

We focused mostly on the utility of PGS to identify individuals at highest risk, defined as those with the 10% highest risk, as opposed to its discriminating power in the remainder of the risk spectrum. We confirm that PGS can be used in a Dutch cohort (Table 1) to identify the top 10% at-risk individuals at an approximately 3.0- and 2.4-fold higher risk of developing T2D and CAD, respectively. However, we also find that individuals that are in the highest risk decile based on BMI, smoking status, physical activity, parental T2D status and sex have an incidence odds ratio of 4.0- and 3.0-fold, compared to the remainder in the Lifelines cohort, for T2D and CAD, respectively (Fig. 2b). This suggests that a risk assessment based on variables that can be obtained through a simple questionnaire or directly from electronic health records are similarly or more accurate than risk prediction based solely on genetics. Due to the ease of attaining such variables, we suggest to continue using the questionnaire approach as a first risk assessment, rather than rely solely on genetic testing to determine risk.

Nonetheless, as genetic testing becomes increasingly more accessible and appealing to individuals, there is a potential to harness this interest to deliver far more accurate risk impressions for numerous preventable chronic conditions. We show and validate in an external cohort that when PGS predictions are augmented with risk factors that can be easily attained through a questionnaire, risk predictions become more accurate improving from approximately a 3.0 and 2.4 fold higher incidence in the top decile to 5.8- and 4.7-fold for T2D and CAD respectively.

Additionally, we showed that PGS-derived risk often does not agree with risk derived from questionnaire-based risk factors (Fig. 4). Our results suggest that many individuals presented with risk assessments solely based on their genetic risk scores, will falsely conclude they are at low or high risk, stressing the need for inclusion of these easily attainable variables into already existing PGS models. As a result, it can occur that an individual feels protected due to a low genetic risk score, despite being at high risk due to being a heavily overweight smoker, when PGS are reported without consideration of other risk factors. As such, it may even be deceiving to report risk based on solely PGS, which is concerning because this is currently often the case with offered PGS services, at least with commercially available genetic tests. Hence, we strongly advocate the inclusion of these additional variables by the commercial parties that readily supply PGS scores to millions of individuals.

Although models based on sex, BMI, parental disease and smoking status perform relatively well, there is still added value of the genetic risk scores, albeit limited, in line with earlier reports^27–29,42^. We observe that when genetic risk is also included on top of sex, BMI, parental disease and smoking status, the incidence odds ratio increases from 4.0 to 5.8 for T2D and from 3.0 to 4.7 for CAD (Fig. 2b). Whether these gains are sufficient to warrant the added cost of a genotyping assessment may, for now, be questionable. However, with the cost of genotyping chips being close to the 30 euro mark and 30X WGS currently periodically being available for less than 200 euro^17^, it is not difficult to imagine that such data will soon be readily available for a large number of individuals. This stresses the need for availability of platforms that allow integrated analysis of genetic and phenotypic data.

Age is still an obvious predictor of prevalence, also in case of T2D, as prevalence is a function of a time. Although we argue that it is unfair to compare disease prevalence of older individuals to younger individuals and should thus not be used in a model that predicts prevalence, this does clearly indicate that age should be considered when presenting individuals with their risk. If age is not considered when informing about risk, the absolute prevalence of a disease may appear irrelevant. For example, an increased prevalence from 0.25% to 2% for diabetes at young ages may appear irrelevant, but when the absolute risk increases at older age from 5 to 40% is communicated, may seem far more substantial and more likely to trigger action. Therefore, it is important to communicate the lifetime risk rather than 10-year risk to individuals of younger ages.

### Role in prevention

The models created in this project can be used to identify individuals at high risk of either CAD or T2D. Depending on the outcome you are at risk for, you may want to take different preventive actions, as different risk factors may be relevant. For instance, high blood pressure can be a risk factor for CAD and can be affected by salt intake. For T2D, high blood pressure is less of an issue, while sugar intake may be much more important to monitor. If an individual is aware of the phenotype they are at highest risk for, they can identify the risk factors that they can reduce to efficiently lower their health risk (as opposed to following all standard guidelines, which cover to wide a range of actions to inspire actual action).

While some individuals are at high genetic risk, which they cannot change, they can still take preventive action to offset their genetic predisposition. Earlier work has indeed shown that those with elevated risk based on genetics can still lower their risk to well below the overweight individual with low genetic risk^43^. Similarly, we observe that individuals with a healthy BMI (between 18.5 and 25) and high genetic risk (top decile), still have a lower or similar incidence than individuals with a high BMI (over 30) and low genetic risk (bottom decile) for T2D or medium genetic risk in case of CAD (Fig. 5). We simultaneously observe that individuals in the highest genetic risk groups stand to gain the most from a healthier lifestyle in terms of reducing risk on an absolute scale. Thus, if a limited number of individuals can be selected for a program to limit or even reduce weight, those in the high genetic risk category should be targeted over those in the low genetic risk category. These predictions can therefore be useful when prevention becomes a more common procedure in health care.

We observe that commercial parties are already playing a relatively big role in preventive health compared to hospitals. We believe this roll will evolve further in the future. From the fact that PGS have already been implemented by numerous companies we can deduce that commercial parties are indeed closely following developments in the scientific field. For this reason, we believe, it is important for the scientific field to guide commercial parties in the right direction and also offer applicable and implementable solutions.

### Limitations

In this paper, we limited our genetic analyses to PGS, which typically do not consider monogenic variants. These are single variants that on their own greatly impact your risk. Iconic examples of such variants are the mutations that occur in *BRCA1* and *BRCA2* genes, which increased risk for breast cancer by more than tenfold^44^. These variants are however rare and despite their large effects typically only explain a small portion, less than 15% of all cases, of the phenotype observed in the population^44^. We observe that the predictive power of easily attainable risk factors is much larger than from PGS. Next to that fact, PGS is reportedly has equivalent predictive power as monogenic analyses^13^, meaning the added cost of WGS, required for appropriate monogenic analyses, is likely not worth it for the purposes described in this paper. We note that genotyping chips are unsuitable for monogenic analysis as the false positive rate for very rare variants, is as high as 5 out of every 6 positives^45^. Furthermore, we note that risk from these monogenic variants is often more difficult if not impossible to mitigate through lifestyle intervention. Lastly, any findings from monogenic mutations would need to be validated and carefully communicated. This is not something easy to implement, which is the aim of this work. While we acknowledge the importance of monogenic analysis in hospital settings, this is not something that warrants the cost for the general public nor should be supplied directly from commercial parties.

There is also a number of ethical limitations to consider when offering polygenic risk scores, which warrant an elaborate dissemination, for which we refer to^46^.

Lastly, in this paper, we focused on T2D and CAD due to their high prevalence, burden on society and their often-preventable nature. We acknowledge that PGS can play a role in screening for other common health conditions as well, such as cancer^13,15,47^ (albeit varying per cancer type^48^), and even rare diseases in the future^49^.

## Conclusion

With the emerging public interest in preventive health^50^, the demand for more personalized risk assessments is likely to keep increasing. While, to some extent, genetic risk profiling is readily commercially available to the general public, most of the reported risk estimations can be greatly improved by using models that include easily accessible variables. To this end, we have developed a SaaS platform that transforms any raw VCF file, independent of the genotyping chip used, into validated risk scores, with the option of taking additional variables such as BMI, sex, age, parental disease and smoking status into consideration to ultimately arrive at more accurate predictions than those available to the public to date. We expect that methods like the one presented here will become commonly used to identify which individuals are at high risk and for what outcome to then be translated into personalised health advice and initiating targeted preventative measures.

## Methods

### Data acquisition and quality control

#### Lifelines data

UMCG Genetics Lifelines Initiative (UGLI) release 1 of the Lifelines genotyping data was used (quality controlled as described in Refs.^51,52^). Additionally, all variants with a minimac3^53^ imputation R^2^ < 0.4 were removed. All variants with more than two alleles were also removed from the data. In addition, all non-Caucasian samples, defined in Ref.^52^ (based on the first 2 PCs), were removed. Additionally, 146 individuals with missing values values for weight, were removed.

After removing these individuals, there were 15 individuals for which the date of diagnoses was missing. After removing these 36,130 individuals remained (Table 1), aged 15–93 (Supplementary Fig. 1).

#### UKB data

Imputed genotyping data for 487,406 individuals were downloaded via protocols provided by the UKB^54^. All variants with an imputation R^2^ < 0.4, according to Ref.^55^, were removed from the data. All variants with more than two alleles were removed from the data. All 78,411 non-Caucasian samples were removed (based on UKB field: 22,006^56^). There were 2715 caucasian individuals with missing values for one or multiple of the variables used in the model: 1973 BMI, 1983 smoking status, 516 high activity, 516 medium activity. After removing these individuals, there were 11 individuals for which the date of diagnoses was missing. They were removed. In total, 406,159 Caucasians remained, aged 38–75.

For both datasets, all NA values for illness of the parents were set to 0 (indicating no illness), instead of removing individuals with NA values for this variable.

#### 1000 genomes (1000g) WGS data

The 1000g phase 3 data^57^ were downloaded and used as a Linkage Disequilibrium (LD) panel, as well as use as a reference to convert PGS scores into percentile/decile scores to allow potential data from other genotyping chips to be put on the same scale (explained in more detail in the supplements).

### PGS score calculation

Polygenic scores are a summation of the effects of multiple, often many, common risk variants. Risk variants and effect sizes are based on GWAS. For the calculation of PGS scores the GWAS summary statistics files were used from 2 different studies. Two from GWAS conducted on European cohorts excluding any of the individuals in the UKB or Lifelines cohorts for T2D^58^, CAD^59^.

From each GWAS summary statistic file two subsets of variants were selected. One containing only those variants with a GWAS significance of 0.01 or more significant. And one containing only those variants with a significance of 10^–6^ or more significant. These subsets were used to calculate one PGS each; two per GWAS summary statistic file. Correlation of each PGS with the respective outcome was calculated and a comparison between the resulting PGS was made. The observation that both the PGS based on a significant cutoff of 0.01 and 10^–6^ performed relatively well, but did not correlate with each other as strongly as we expected (Supplementary Fig. 4), suggesting that combining both scores would potentially yield a PGS that predicts the outcome better. For this reason, the PGS resulting from the two separate analyses using the 2 different cutoffs were summed to ultimately arrive at the PGS used in this paper (Supplementary Fig. 2).

Any genetic variants that were not present in all three files (GWAS summary statistic, UKB/Lifelines genotyping or the 1000g WGS reference data), were removed from the data prior to subsequent analyses.

LDpred version 1.0.11 was used to calculate PGS scores, using the 1000g WGS data^57^ as reference LD panel to calculate the posterior mean of the effect sizes under an infinitesimal model. In other words, the GWAS variant effect sizes were reweighed based on their LD with other variants. Only LDpred-inf scores were calculated to optimize the speed of the analysis.

LDpred-inf PGS scores were calculated for UKB, Lifelines and the 1000g individuals. The UKB and Lifelines scores were converted into a score from 1 to 100 based on the percentile they would be in in the PGS score distribution of the 503 European 1000g individuals. This is necessary since Lifelines and UKB are based on different genotyping chips with only 1/3 overlap in the genetic variants measured. As a consequence, the distribution of the resulting absolute PGS scores do not overlap; This problem is solved by calibrating them to the 503 European 1000g individuals as described above. For each GWAS summary static file the two PGS scores calculated were summed, referred to as summed PGS.

### Prediction models

To calculate the c-indexes using different prediction variables, the following model was used or a subset thereof (for a full list of models used we refer to the supplements):

$$\mathrm{Model }Outcome \sim BMI+Smoker+PA+Sex+Parent+PGS+Age+PGS*BMI,$$

$$logit\left({p}_{i}\right)={\upbeta }_{0}+{\upbeta }_{bmi}BM{I}_{i}+{\upbeta }_{smokerPast}SmokerPas{t}_{i}+{\upbeta }_{smokerCurrent}SmokerCurren{t}_{i}+{\upbeta }_{physicalActivityModerate}PhysicalActivityModerat{e}_{i}+{\upbeta }_{physicalActivityVigorous}PhysicalActivityVigorou{s}_{i}+{\upbeta }_{sex}Se{x}_{i}+{\upbeta }_{fatherDiseaseStatus}FatherDiseaseStatu{s}_{i}+{\upbeta }_{motherDiseaseStatus}MotherDiseaseStatu{s}_{i}+{\upbeta }_{pgs}PG{S}_{i}+{\upbeta }_{age}Ag{e}_{i}+{\upbeta }_{PgsBmi}PG{S}_{i}*BM{I}_{i}+\sum_{pc=1}^{4}{\upbeta }_{pc} P{C}_{i}+\sum_{batch=1}^{95}{\upbeta }_{batch} batc{h}_{i},$$

where β_0_ is the intercept, and β_bmi_, β_smokerPast_, β_SmokerCurrent_, β_physicalActivityModerate_, β_physicalActivityVigorous_, β_sex_, β_fatherDiseaseStatus_, β_motherDiseaseStatus_, β_pgs,_ β_age_ the regression coefficient for the respective variables and β_PgsBmi_ the regression coefficient for the multiplicative term. β_pc_ is the regression coefficient for the respective PC and β_batch_ the regression component for the respective batch. *Outcome* is the diagnosis status for either T2D or CAD, described in the supplementary information. Annotations for each outcome are annotated in Supplementary Table 2.

Separate models were constructed to predict prevalence and incidence. The model coefficients were calculated based on the training set, consisting of the UK Biobank, which were subsequently applied to the Lifelines dataset used for testing. We did not use any dataset to optimize which parameters should be included in the model. The motivation for this choice was that we had a predetermined set of variables we aimed to test and wanted to avoid potential overfitting issues that could potentially translate to poor performance in the Lifelines dataset used for testing.

The predictions models for incidence were built and tested using the same approach, but on a subset of the data from which individuals that had obtained the outcome before their initial measurement were removed.

### Relative risk calculations

A Cox regression model was fit on the UK Biobank data and using the resulting predictor coefficients were applied to the Lifelines dataset to attain a prediction value for each individual in this dataset. To determine the odds ratio of the top decile against the remaining deciles, a logistic regression was fit onto these scores calculated based on the Cox model. Coefficients of the fit logistic model were exponentiated to calculated the odds ratio of the relevant predictor in the model (Fig. 2b). Statistical difference between the odds ratios of different models is determined using a likelihood ratio test, indicating whether there is a statistically significant difference between the probability that is assigned to each individual in relation to their actual outcome.

All methods were carried out in accordance with relevant guidelines and regulations (see ethical approval and Data and code availability).

### SaaS platform

Additionally, we have constructed a SaaS platform that can perform assessments on single genotyping chips, to determine an individual's risk based on the observed prevalence and incidence of a number of outcomes, among which T2D and CAD reported in this paper. This includes a number of quality control steps already applied to the data supplied by the UKB and Lifelines. These steps are further explained in the supplements.

### Ethical approval

UK Biobank has approval from the North West Multi-centre Research Ethics Committee (MREC) as a Research Tissue Bank (RTB) approval. This approval means that researchers do not require separate ethical clearance and can operate under the RTB approval. The Lifelines protocol was approved by the UMCG Medical ethical committee under number 2007/152. All participants signed an informed consent form. No participants were re-contacted during this project.

## Supplementary Information

Supplementary Information.

## Data Availability

All results and code created during this project are available upon request, if sharable in accordance with the UK Biobank and Lifelines material transfer agreement, by contacting the corresponding author of this paper (Sipko van Dam). We adhered to the 'Scientific Reports' policies on sharing data and materials. The manuscript is based on data from the UK Biobank through application 55495. The Resource is available to all bona fide researchers for all types of health-related research that is in the public interest, without preferential or exclusive access for any person. The catalogue of the UK Biobank is accessible at https://biobank.ndph.ox.ac.uk/ukb/catalogs.cgi. All international researchers can obtain data access at https://www.ukbiobank.ac.uk/enable-your-research/apply-for-access. A fee is required. The manuscript is based on data from the Lifelines Cohort Study, Study OV20_00020. Lifelines adheres to standards for data availability. Due to ethical restrictions imposed by the Lifelines Scientific Board and the Medical Ethical Committee of the University Medical Center Groningen related to protecting patient privacy, the data are not publicly available. The data catalogue of Lifelines is publicly accessible at http://www.lifelines.net. All international researchers can obtain data at the Lifelines research office (research@lifelines.nl), for which a fee is required. The Lifelines and UK Biobank systems allow access for reproducibility of the study results.
