# Supplementary material for: The necessity of incorporating non-genetic risk factors into polygenic risk score models
Source: Sci Rep. 2023 Feb 20;13:1351. doi: 10.1038/s41598-023-27637-w (PMC9941118; doi:10.1038/s41598-023-27637-w)
Supplement: Supplementary file 1 — Supplementary Information. [file 41598_2023_27637_MOESM1_ESM.docx]

## Supplementary notes

## PGS method selection and validation

While we aimed to use the most optimal methods to predict PGS, we originally noticed different papers inconsistently report varying optimal PGS strategies. These methods vary from simple yet effective clumping and thresholding using PRSice-2 ^1^, stacked clumping and thresholding (SCT) ^2^, to other methods such as, Lassosum ^3^, Ldpred-2 ^4^, SbayesR ^5^, PRS-CS and more ^6^, all predicting Type-2 diabetes (T2D) using UK Biobank (UKB) data. Furthermore, it was shown the optimal method can also vary on environment of the population on which the GWAS input files were generated as well as the target population in which the PGS are predicted ^7^. In an independent extensive comparison, it was shown that the best model varies per phenotype ^6^.

As such, we originally set out to first calculate PGS using a simple computationally less intensive method (ldpred-inf) using a limited set of SNPs. We compared two analyses to compare how the inclusion of more or less SNPs would affects the predictive performance. These two analyses varied only in that they included a different set of input SNPs based on two arbitrarily selected different Genome Wide Association Study (GWAS) significance cutoffs: 10^-6^ and 0.01. We found that both translate into relatively similar biologically meaningful results in terms of identifying individuals at higher risk (Supplementary figure 4). Early reported PGS performance however was slightly worse than reported in current literature, reporting a 2.9 and 2.5 fold increased risk for the individuals in the highest decile as compared to the rest, for T2D and CAD ^8^ as opposed to 2.4 and 2.2 fold in our exploratory analysis.

In addition to the performance of our PGS predictions on an overall level, we also assessed the robustness of these two different PGS on a per user level. We compared the risk percentiles each individual is part of based on the 2 PGS calculated using the 2 different GWAS significance thresholds. We found that the results on a per person level can greatly differ, while c-indexes using these different cutoffs only differed marginally. We found that the Pearson correlation between the predicted risk percentiles of these two PGS was 0.36 for T2D and 0.37 for CAD in the UKB.

We concluded that, if the predictive power of PGS at both cutoffs is similar but the individual results differ, that both PGS lists harbor different information. As such, a combined model based on both PGS scores should result in more accurate predictions. Indeed, we observe that a model based on both PGS combined finds has an increased C-index compared to the best performing one of either separate, with the C-indexes of the different models being as follows:

T2D:

Predicting prevalence:

c-index 0.01 p-value cutoff: 0.615 (95 % confidence interval: 0.612-0.619)

c-index 10^-6^ p-value cutoff: 0.604 (95 % confidence interval: 0.600-0.608)

c-index combined: 0.630 (95 % confidence interval: 0.627-0.634)

Predicting incidence:

c-index 0.01 p-value cutoff: 0.589 (95 % confidence interval: 0.583-0.596)

c-index 10^-6^ p-value cutoff: 0.583 (95 % confidence interval: 0.576-0.591)

c-index combined: 0.602 (95 % confidence interval: 0.597-0.608)

CAD:

Predicting prevalence:

c-index 0.01 p-value cutoff: 0.62 (95 % confidence interval: 0.616-0.626)

c-index 10^-6^ p-value cutoff: 0.596 (95 % confidence interval: 0.592-0.601)

c-index combined: 0.629 (95 % confidence interval: 0.626-0.634)

Predicting incidence:

c-index 0.01 p-value cutoff: 0.593 (95 % confidence interval: 0.588-0.598)

c-index 10^-6^ p-value cutoff: 0.579 (95 % confidence interval: 0.573-0.585)

c-index combined: 0.604 (95 % confidence interval: 0.599 -0.611)

The outcome prevalence odds ratios for individuals in the highest risk decile according to the combined model are 2.9 and 2.5 (supplementary figure 2, supplementary table 1), for T2D and CAD respectively, compared to the also reported approximate odds ratio of 2.5 and 2.9 fold for incidence and prevalence combined, reported earlier in literature ^8,9^. Hence our models were able to identify individuals at high risk similarly, even though we corrected for additional covariates including, BMI, parental disease status, physical activity and smoking status. We initially chose our approach as a quick and simple test but found that after combining the results for 2 different thresholds, the performance was similar to that of far more computationally intense methods ^8^ and decided to use our approach, but acknowledge that there is likely room for further minor improvements trying different strategies, albeit at the cost of introducing some bias due to multiple testing.

As also reported previously^8,9^, we found that individuals in the highest decile have a substantially higher risk of the respective disease and see these are consistent across different ages in the UK Biobank (Figure2, supplementary figure 2, supplementary table 1). Risk estimated based on PGS calculated on the Lifelines cohort similarly reveal a risk increase for the highest decile (Figure 2, supplementary table 1). However, the confidence interval is larger in Lifelines, likely due to the much smaller sample size and larger age range. Additionally, AUROCs were computed (Figure 2, supplementary table 1).

All models were first trained and tested for the prediction of prevalence. When calculating the coefficients for prediction components for the prevalence, we corrected the data for the following variables: genetic sex, weight, physical activity and smoking status, parental disease status, age PC_1,2,3,4_ and genotyping measurement batch. When testing, the same covariates were included, provided they were not already included as a predictor in the model under question. C-indexes were calculated based on the test data using the disease annotation as listed below.

The following models were used:

Model $Outcome \sim PGS$

$$logit(p_{i})=\beta_{0}+\beta_{pgs}PGS_{i}+\sum_{covariate=1}^{n} \beta_{covariate}Covariate_{i}$$

Model $Outcome \sim PGS+Age+Sex$

$$logit(p_{i})=\beta_{0}+\beta_{pgs}PGS_{i}+\beta_{age}Age_{i}+\beta_{sex}Sex_{i}+\sum_{covariate=1}^{n} \beta_{covariate}Covariate_{i}$$

Model $Outcome \sim BMI$

$$logit(p_{i})=\beta_{0}+\beta_{bmi}BMI_{i}+\sum_{covariate=1}^{n} \beta_{covariate}Covariate_{i}$$

Model $Outcome \sim BMI+Smoker$

$$logit(p_{i})=\beta_{0}+\beta_{bmi}BMI_{i}+\beta_{smokerPast}SmokerPast_{i}+\beta_{smokerCurrent}SmokerCurrent_{i}+{\sum_{covariate=1}^{n} \beta_{covariate}Covariate}_{i}$$

Model $Outcome \sim BMI+Smoker+PA$

$$logit(p_{i})=\beta_{0}+\beta_{bmi}BMI_{i}+\beta_{smokerPast}SmokerPast_{i}+\beta_{smokerCurrent}SmokerCurrent_{i}+\beta_{physicalActivityModerate}PhysicalActivityModerate_{i}+\beta_{physicalActivityVigorous}PhysicalActivityVigorous_{i}+\sum_{covariate=1}^{n} \beta_{covariate}Covariate_{i}$$

Model $Outcome \sim BMI+Smoker+PA+Sex$

$$logit(p_{i})=\beta_{0}+\beta_{bmi}BMI_{i}+\beta_{smokerPast}SmokerPast_{i}+\beta_{smokerCurrent}SmokerCurrent_{i}+\beta_{physicalActivityModerate}PhysicalActivityModerate_{i}+\beta_{physicalActivityVigorous}PhysicalActivityVigorous_{i}+\beta_{sex}Sex_{i}+\sum_{covariate=1}^{n} \beta_{covariate}Covariate_{i}$$

Model $Outcome \sim BMI+Smoker+PA+Sex+Parent$

$$logit(p_{i})=\beta_{0}+\beta_{bmi}BMI_{i}+\beta_{smokerPast}SmokerPast_{i}+\beta_{smokerCurrent}SmokerCurrent_{i}+\beta_{physicalActivityModerate}PhysicalActivityModerate_{i}+\beta_{physicalActivityVigorous}PhysicalActivityVigorous_{i}+\beta_{sex}Sex_{i}+\beta_{fatherDiseaseStatus}FatherDiseaseStatus_{i}+\beta_{motherDiseaseStatus}MotherDiseaseStatus_{i}+\sum_{covariate=1}^{n} \beta_{covariate}Covariate_{i}$$

Model $Outcome \sim BMI+Smoker+PA+Sex+Parent+PGS$

$$logit(p_{i})=\beta_{0}+\beta_{bmi}BMI_{i}+\beta_{smokerPast}SmokerPast_{i}+\beta_{smokerCurrent}SmokerCurrent_{i}+\beta_{physicalActivityModerate}PhysicalActivityModerate_{i}+\beta_{physicalActivityVigorous}PhysicalActivityVigorous_{i}+\beta_{sex}Sex_{i}+\beta_{fatherDiseaseStatus}FatherDiseaseStatus_{i}+\beta_{motherDiseaseStatus}MotherDiseaseStatus\_i+\sum_{covariate=1}^{n} \beta_{covariate}Covariate_{i}$$

Model $Outcome \sim BMI+Smoker+PA+Sex+Parent+PGS+Age$

$$logit(p_{i})=\beta_{0}+\beta_{bmi}BMI_{i}+\beta_{smokerPast}SmokerPast_{i}+\beta_{smokerCurrent}SmokerCurrent_{i}+\beta_{physicalActivityModerate}PhysicalActivityModerate_{i}+\beta_{physicalActivityVigorous}PhysicalActivityVigorous_{i}+\beta_{sex}Sex_{i}+\beta_{fatherDiseaseStatus}FatherDiseaseStatus_{i}+\beta_{motherDiseaseStatus}MotherDiseaseStatus_{i}+\beta_{pgs}PGS_{i}+\beta_{age}Age_{i}+\sum_{covariate=1}^{n} \beta_{covariate}Covariate_{i}$$

Full model:

Model $Outcome \sim BMI+Smoker+PA+Sex+Parent+PGS+Age+PGS*BMI$

$$logit(p_{i})=\beta_{0}+\beta_{bmi}BMI_{i}+\beta_{smokerPast}SmokerPast_{i}+\beta_{smokerCurrent}SmokerCurrent_{i}+\beta_{physicalActivityModerate}PhysicalActivityModerate_{i}+\beta_{physicalActivityVigorous}PhysicalActivityVigorous_{i}+\beta_{sex}Sex_{i}+\beta_{fatherDiseaseStatus}FatherDiseaseStatus_{i}+\beta_{motherDiseaseStatus}MotherDiseaseStatus_{i}+\beta_{pgs}PGS_{i}+\beta_{age}Age_{i}+\beta_{PgsBmi}PGS_{i}*BMI_{i}+\sum_{pc=1}^{4} \beta_{pc} PC_{i}+\sum_{batch=1}^{95} \beta_{batch} batch_{i}$$

Where β_0_ is the intercept, and β_pgs_, β_sex_, β_age_, β_bmi_, β_smoker_, β_pa_, β_sex_, β_parent_ the regression coefficient for the respective variables and β_PgsBmi_  the regression coefficient for the multiplicative term. β_pc_ is the regression coefficient for the respective PC and β_batch_ the regression component for the respective batch. β_covariates_ details the regression coefficients for the included respective covariate. In all cases, variables not included as predictors are included as covariates.

*Outcome* is the diagnosis status for either T2D or CAD, described in more detail below (section "Disease annotation").

## Disease annotation

The models were built to predict either T2D or CAD. These annotations were defined depending on the biobank's available information as described below.

**UKBiobank**

Disease annotations were based on self-report in an interview with a trained nurse (Data-Fields 20002 and 20004) or via ACE touchscreen questions (Data-Fields 2443 and 6150), hospital reports coded according to the International Classification of Disease version 9 (ICD-9, Data-Field 41271) and version 10 (ICD-10, Data-Field 41270), and reported operative procedures coded according to the Office of Population Censuses and Surveys Classification of Interventions and Procedures version 4 (OPCS-4, UKB Data-Field 41272).

Supplementary table 2 lists all the Data-Fields used to calculate disease incidence and the Data-Fields of the associated ages or dates of diagnosis that were used to determine if participants developed the disease before or after their first UKB center visit.

For CAD, diagnosis was based on self-reported heart attack (Data-Fields 20,002), heart attack diagnosed by doctor (Data-Field 6150), self-reported operation (PTCA, CABG or triple heart bypass, Data-Field 20,004), hospital admission for myocardial infarction (ICD-9 codes 410.X, 411.0, 412.X and 429.79, Data-Field 41,271, and ICD-10 codes I21.X, I22.X, I23.X, I24.X, I25.2, Data-Field 41,270), reported coronary artery bypass grafting (OPCS-4 codes K40.1–40.4, K41.1–41.4, or K45.1–45.5, Data-Fields 41272) and reported coronary angioplasty (OPCS-4 codes K49.1–49.2, K49.8–49.9, K50.2, K75.1–75.4, and K75.8–75.9, Data-Fields 41,272 and 41,200).

T2D diagnoses were based on diabetes diagnosed by doctor (Data-Field 2,443), self-reported type 2 diabetes (Data-Field 20,002), type 2 diabetes hospital record annotation (ICD-9 codes 250.X0, 250.X2, Data-Field 41,271 and ICD-10 codes E11.X, Data-Field 41,270). Individuals with self-reported T1D were annotated as not having T2D.

### **Lifelines**

### Disease annotations for Lifelines were based on questionnaires the participants filled in on paper. Questionnaire data from assessment 1A was used to determine if participants were diagnosed with the disease before the first measurement. All other assessment questionnaires (1B, 1C, 2A, 2B, 2C, 3A) were used to annotate if participants developed the disease after the first measurement.

### CAD diagnosis was based on self-reported heart attack (code heartattack_presence_adu_q_1 for 1A and code infarction_followup_adu_q_1 for 1B, 1C, 2A and 3A) and self-reported angioplasty and/or bypass surgery (code angioplasty_bypass_adu_q_1 for 1A and 3A).

T2D diagnosis was based on self-reported type 2 diabetes (codes diabetes_type_adu_q_1 and diabetes_type_adu_q_2 for 1A and code t2d_followup_adu_q_1 for 2A and 3A)

# SaaS platform to estimate risk for single genotyping chips

To support implementation of the scientific methods described in this paper for use in practice, we have constructed a SaaS platform that allows translation of genotyping files into risk scores for chronic diseases, such as T2D and CAD.

The SaaS platform requires either a VCF, bgen or pgen file as input and performs the following steps to finally arrive at risk estimates for a particular individual based on their genetic profile (Age, sex and gender information can be added to arrive at vastly more accurate predictions).

1. Where necessary SNPs are flipped to match the reference genome (1000g)
2. Poor quality samples are removed (>2.5% missing genotype calls)
3. Poorly genotyped SNPs are removed (>2.5% missing genotype calls)
4. Genotypes with a Hardy Weinberg Equilibrium p-value below 10^-6^ are removed
5. Samples with a high heterozygosity rate (4 standard deviations of the mean of the reference panel) are removed
6. Samples are imputed against the Haplotype Reference Consortium (r1.1) panel, using the Sanger imputation service ^10^
7. Sex is determined based on genotyping data.
8. PGS scores are calculated as described in the method section of the paper.

To allow for calculation of PGS, it is possible to conduct a GWAS on the UK Biobank data for any trait that is available in both the UK Biobank and Lifelines data. PGS will be calculated on the UK Biobank cohort and the predictive power is computed on the Lifelines data.

## PGS can only be translated into risk scores by comparing to a reference cohort using the same genotyping SNPs

Although PGS can be calculated for any individual, they are nothing more than an arbitrary number on their own. To assign a risk to a PGS of an individual the score is normally compared to a reference distribution of PGS ^8^. With such analyses it is important that the reference PGS are calculated in the same manner using the same list of input SNPs. Here, we are faced with a challenge that the single genetic profile is not necessarily based on the same genotyping chip as the reference cohort.

Since different genotyping chips often contain different SNPs, this can lead to issues, especially when there is a need to calculate a risk score for a single genotyping sample that is based on a different genotyping chip compared to any reference cohort available. For example, the overlap between genetic variants measured by the Global Screen Array (GSA) chip used in Lifelines and the UK Biobank (UKB) Axiom Array chip is only 34%. The raw PGS scores calculated based on these two different chips clearly follow different distributions, where both the diabetes, as well as the other PGS scores for the Lifelines diabetics are greatly differing from both the diabetics, as well as the other PGS scores in the UK Biobank (Figure 3)(p-value < 10^-323^, Mann-Whitney U test) and would normally incorrectly suggest most of the, in this example, Lifelines individuals are at high risk for diabetes if they were compared to the UK Biobank PGS distributions. To allow assigning a risk to a single genotyping SNP, for which only a reference cohort based on a different genotyping chip is available, we have therefore devised a method that allows a score based on this chip to be placed in the perspective of any cohort.

To be able to compare results from a different type of genotyping chip, we used an intermediary cohort for which WGS data is available. This WGS data will contain SNP data from both genotyping chips. To do this we used the European individuals in the 1000genomes (1000g) data. This is a dataset containing WGS data for 2500 individuals of which 503 of European descent. Unfortunately, for the 1000g data not all phenotype information is available, making it impossible to assess for each phenotype what risk is associated to the different scores identified. However, we can determine the percentile the individual ranks in compared to the 1000g data. We observed that the distributions for diabetics between both cohorts largely overlap using this method (p-value: 0.76, two sided Mann-Whitney U test t-test) (Supplementary figure 7, left). For the non-diabetics this is also the case albeit there still is a small, yet statistically significant difference between the two distributions (p-value: 1.6 x 10^-35^, two sided Mann-Whitney U test t-test).

Then we can assess the corresponding incidence based on the incidence of individuals in the reference cohort, which in this case could be either the UK Biobank non-diabetics, or the Lifelines non-diabetics. Doing so, we observe similar risk increases in both cohorts (Table 1).

In terms of predicted absolute risk, we did observe a difference, which we can attribute to the overall lower incidence of diabetes in the Lifelines cohort, due to its much lower average age. Correcting for this, yields also similar absolute risk predictions. Using this method, we find that relative risk is comparable in both cohorts and in line with earlier findings ^10,11^, showing that this method is robust. As such this method can be used to assess the risk of single individuals, without the need for the use of the same genotyping chip.

This is particularly of interest since PGS currently suffer from a bias toward Europeans ^11^. Although new biobanks for different ethnicities will resolve the accessibility of reference genotyping chips of the appropriate ethnicity, these will likely contain different genotypes. One drawback that will remain is that for subjects with a complex history of admixture, there will likely be additional challenges, but deconvolution, based on the ethnicity of origin, of such genomes suggests that also for these individuals it will be possible to calculate accurate PGS provided references for the deconvoluted genomes are available ^12^. Therefore, our method will allow any individual chip to be compared against the dataset with most appropriate for that ethnicity when such database become available in the future.

# Supplementary figures


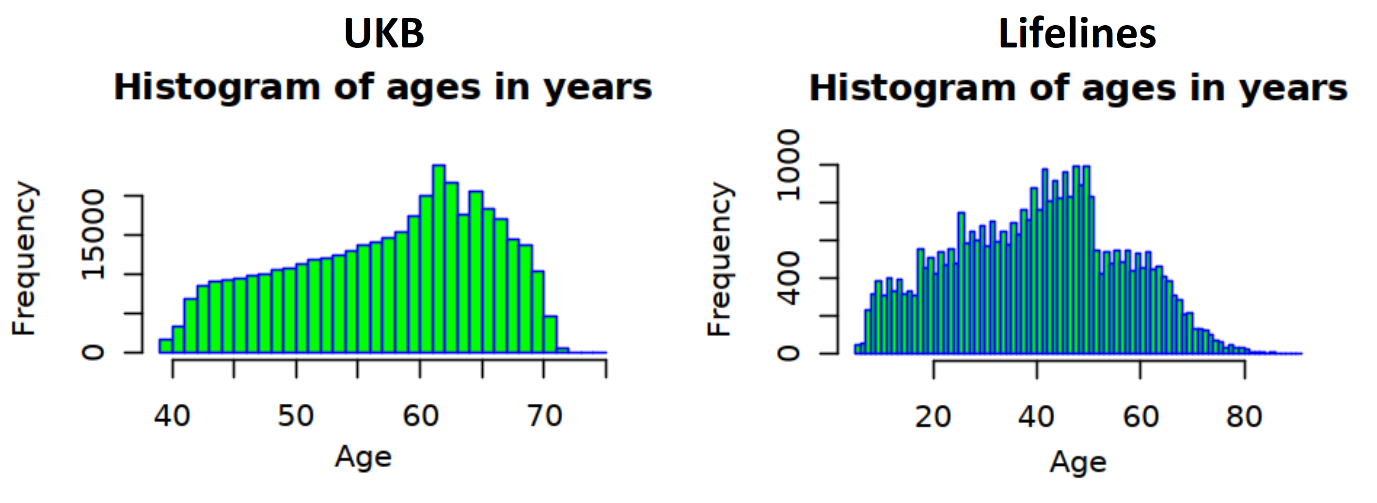


**Supplementary figure 1: Ages of individuals in the biobanks.** Left Lifelines, right UK Biobank.


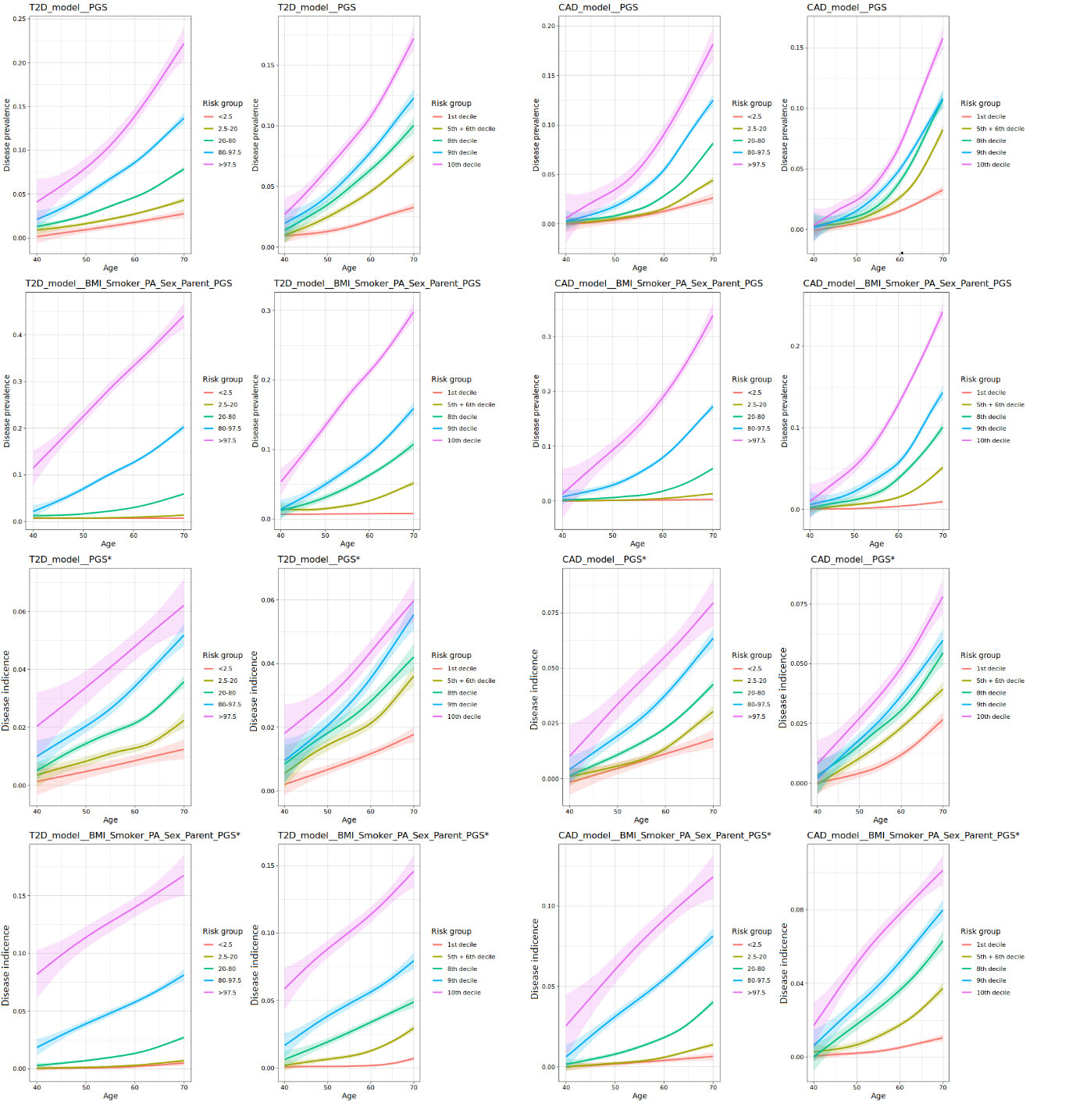


Supplementary figure 2. Comparison of risk in different strata at different ages. Top 2 rows: prevalence. Bottom to rows: incidence. Risk strata were selected to allow comparison against earlier work ^13^.


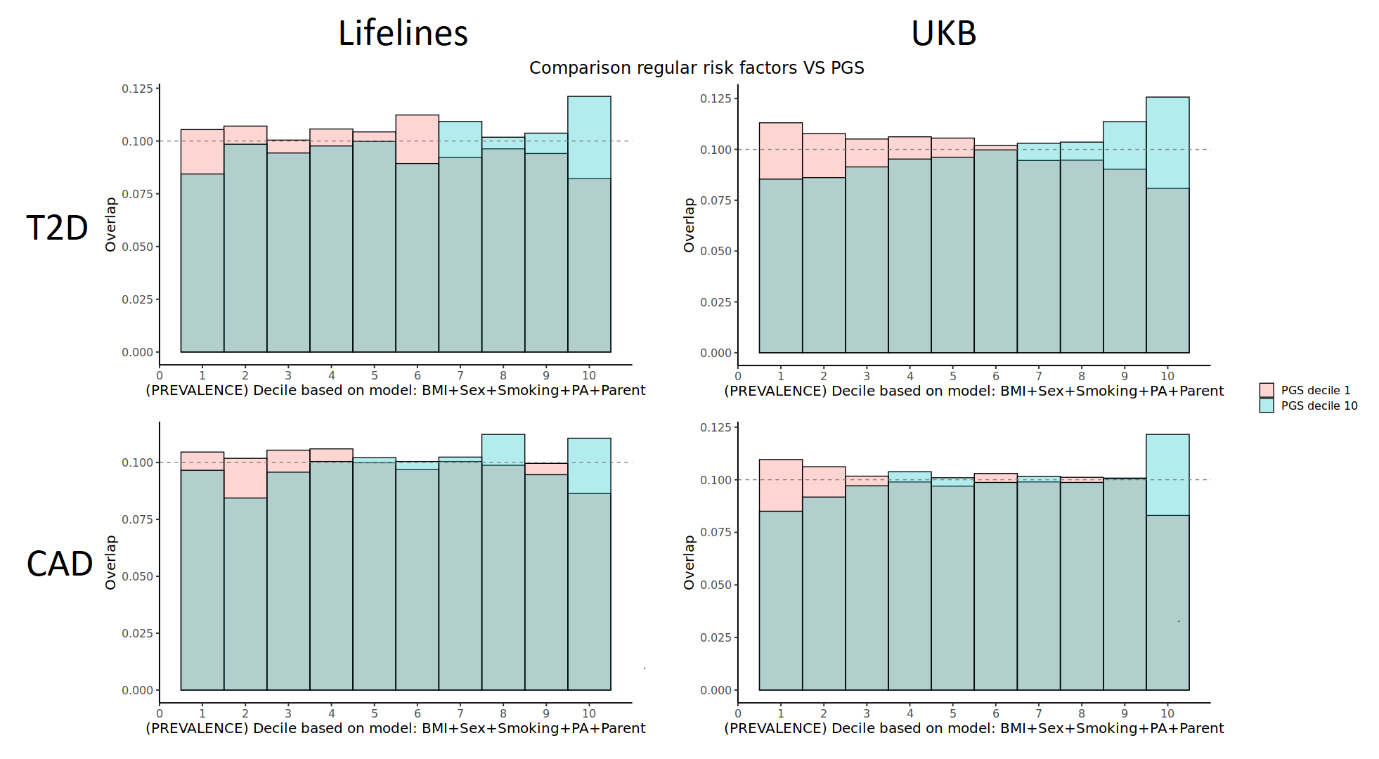


Supplementary figure 3: Comparison outcome prevalence predictions based on a model based on PGS and a model based on Sex, BMI, physical activity, parental T2D disease and Smoking status in both Lifelines (figure 2) and the UK Biobank. Correlation in the UKB is 0.06 (p-value < 2.5*10^-324^) and 0.04 (p-value: 1.4 *10^-132^) with 61% and 63% of the individuals being predicted at least 3 deciles apart from each other, for T2D and CAD respectively. Correlation in the Lifelines is 0.04 (p-value: 2.5 *10^-14^) and 0.03 (p-value: 3.2 *10^-07^) with 63% and 63% of the individuals being predicted at least 3 deciles apart from each other, for CAD and T2D respectively.

Ukb – UK Biobank


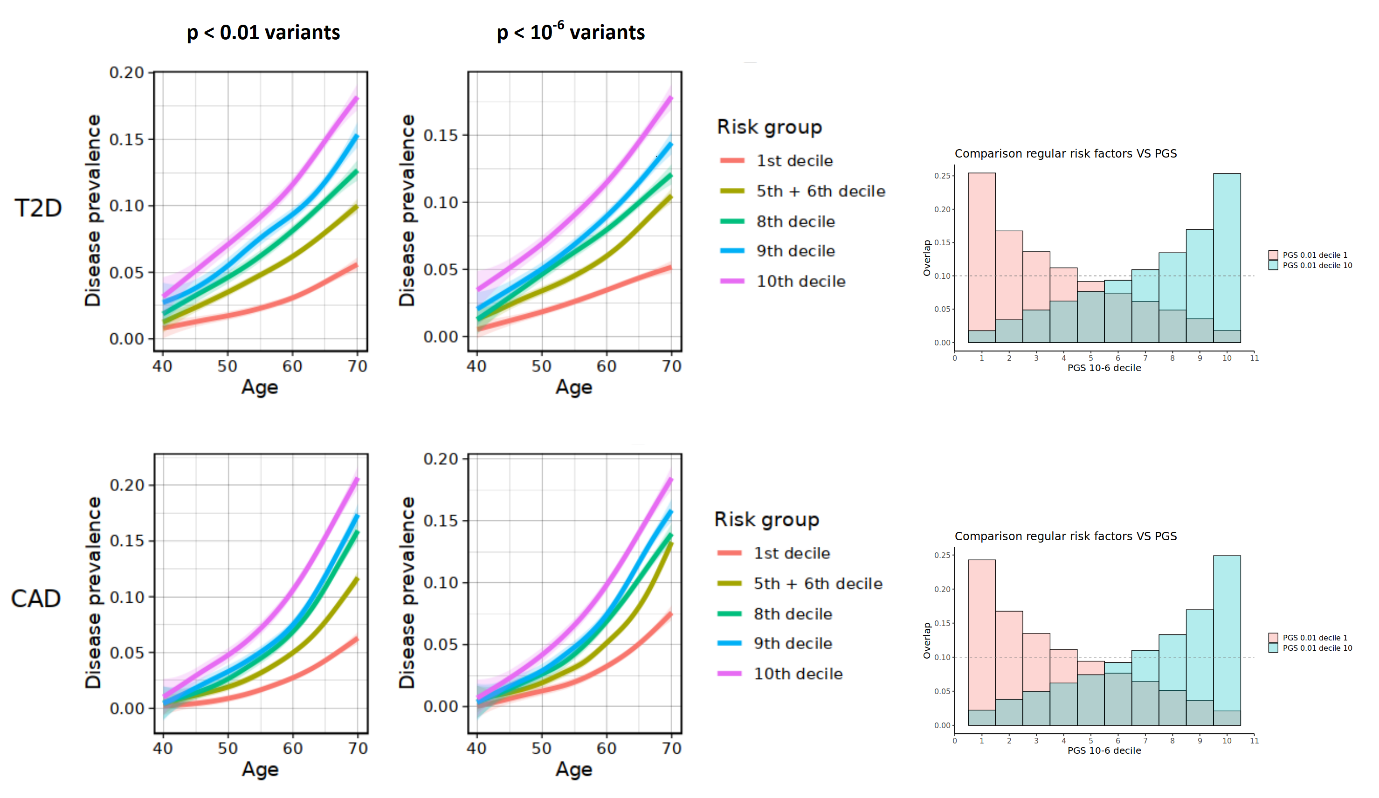


Supplementary figure 4. Comparison of predictions of top and bottom decile from PGS analyses calculated using different prediction models in the UK Biobank.

Left: Prevalence in different risk deciles based on scores calculated using 2 different sets of input SNPs. Either all variants with GWAS significance < 0.01 or all variants with GWAS significance p-value < 10^-6^ were included. Both scores perform relatively well.

Right: The overlap between the individuals predicted in the highest and lowest risk decile according to PGS calculated using a more limited list of SNPs and a more elaborate list based on GWAS significance cutoffs 0.01 and 10^-6^, is lower than we had expected, indicating that even this single different setting can have a great impact on the risk predictions on a personal level. Correlation between the 2 PGS calculation settings is 0.36 and 0.35 in Lifelines and 0.35 and 0.35. In the UKB for T2D and CAD respectively, with a 38-39% of the individuals being classed 3 deciles or more differently in Lifelines and 38-39% in the UKB.

Supplementary tables

Supplementary tables 1: Comparison of models including only PGS or additional variables that can be attained through a questionnaire. Pval column indicates the significance of the difference of the prevalence/incidence of individuals in the top decile compared to the 5^th^ and 6^th^ decile. AllIndividuals column indicates whether the line pertains to predicting incidence or prevalence.

Lifelines model C-indexes:


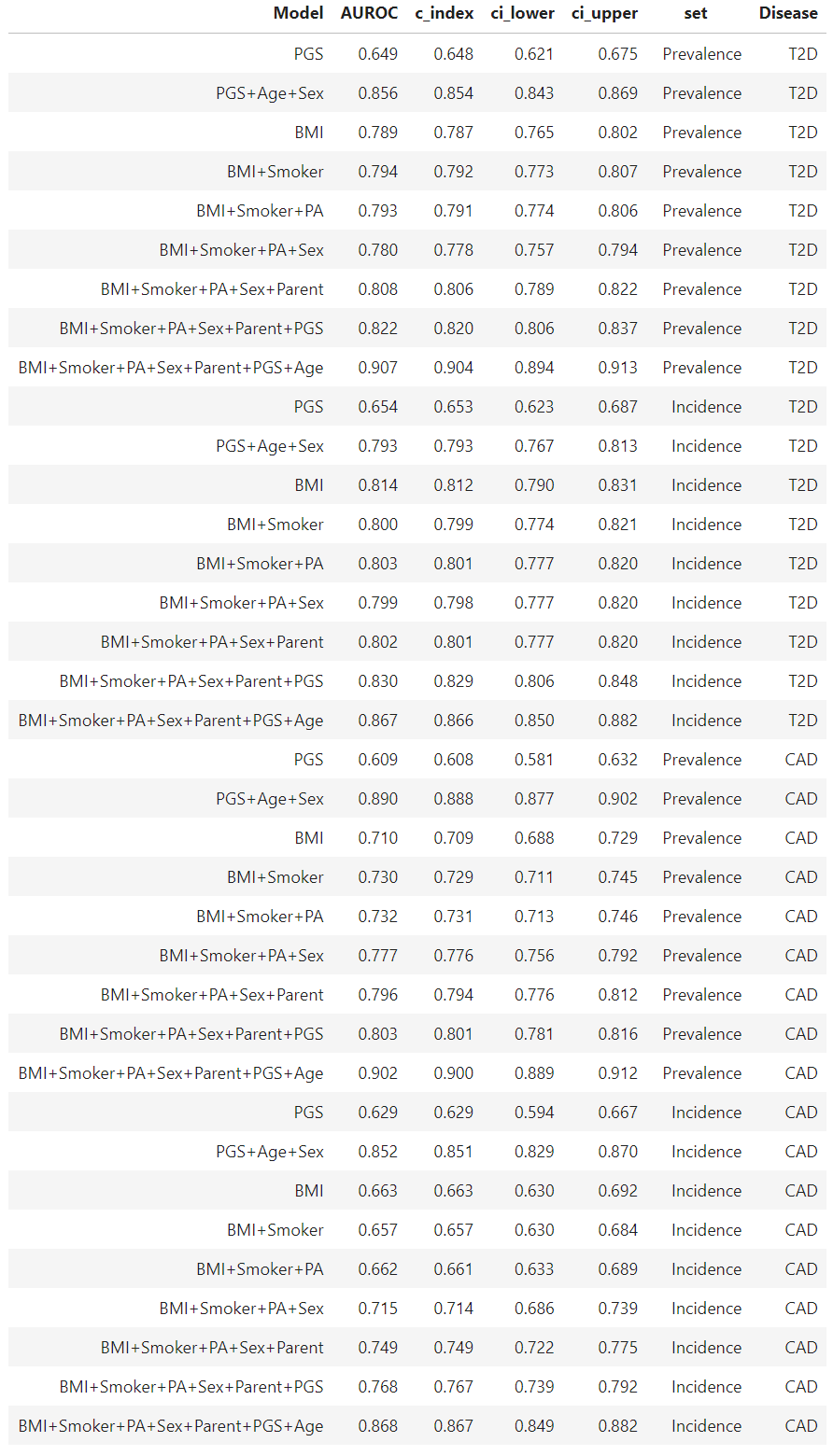


Lifelines odds ratios top decile: **
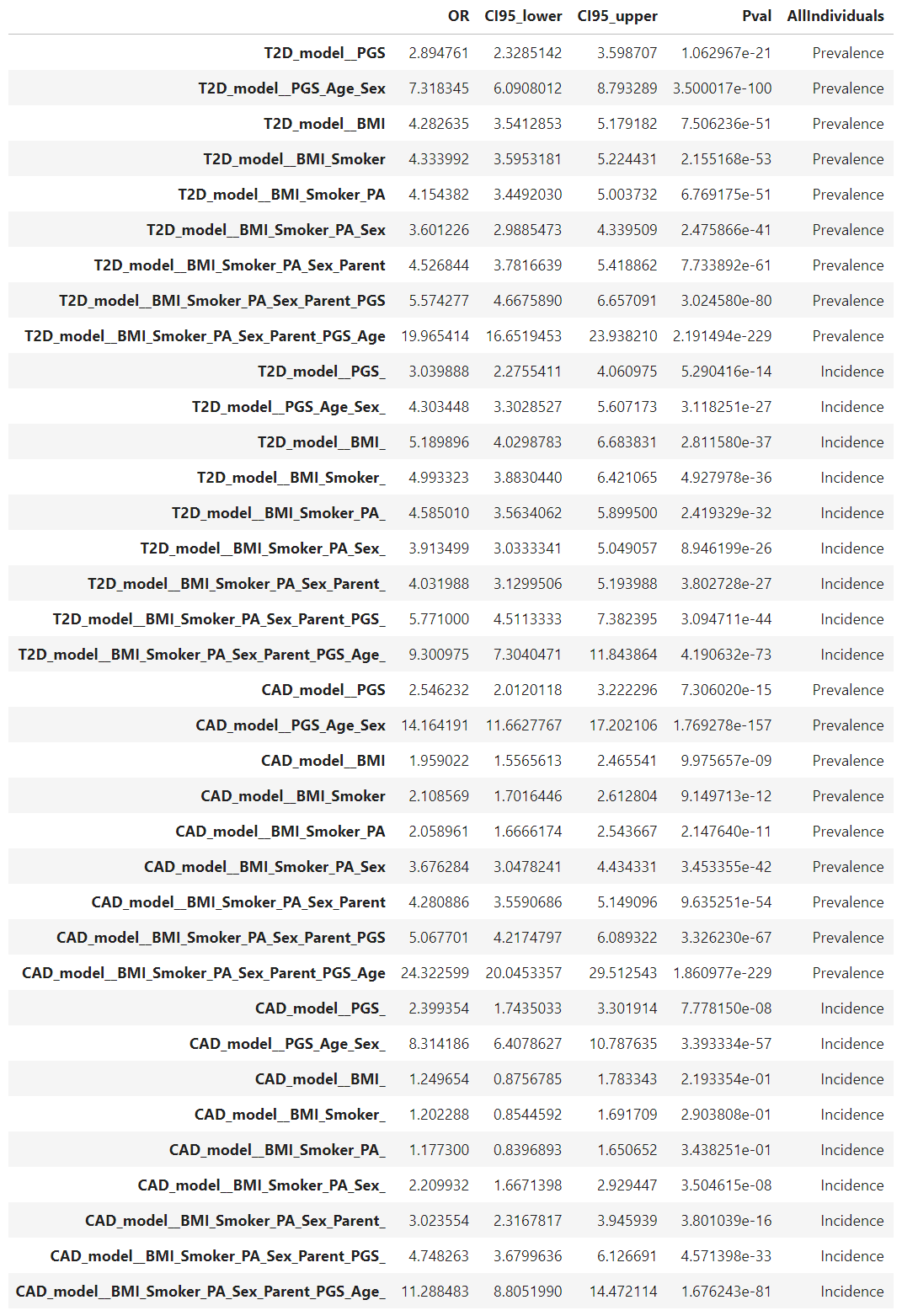
**

UKB model C-indexes:
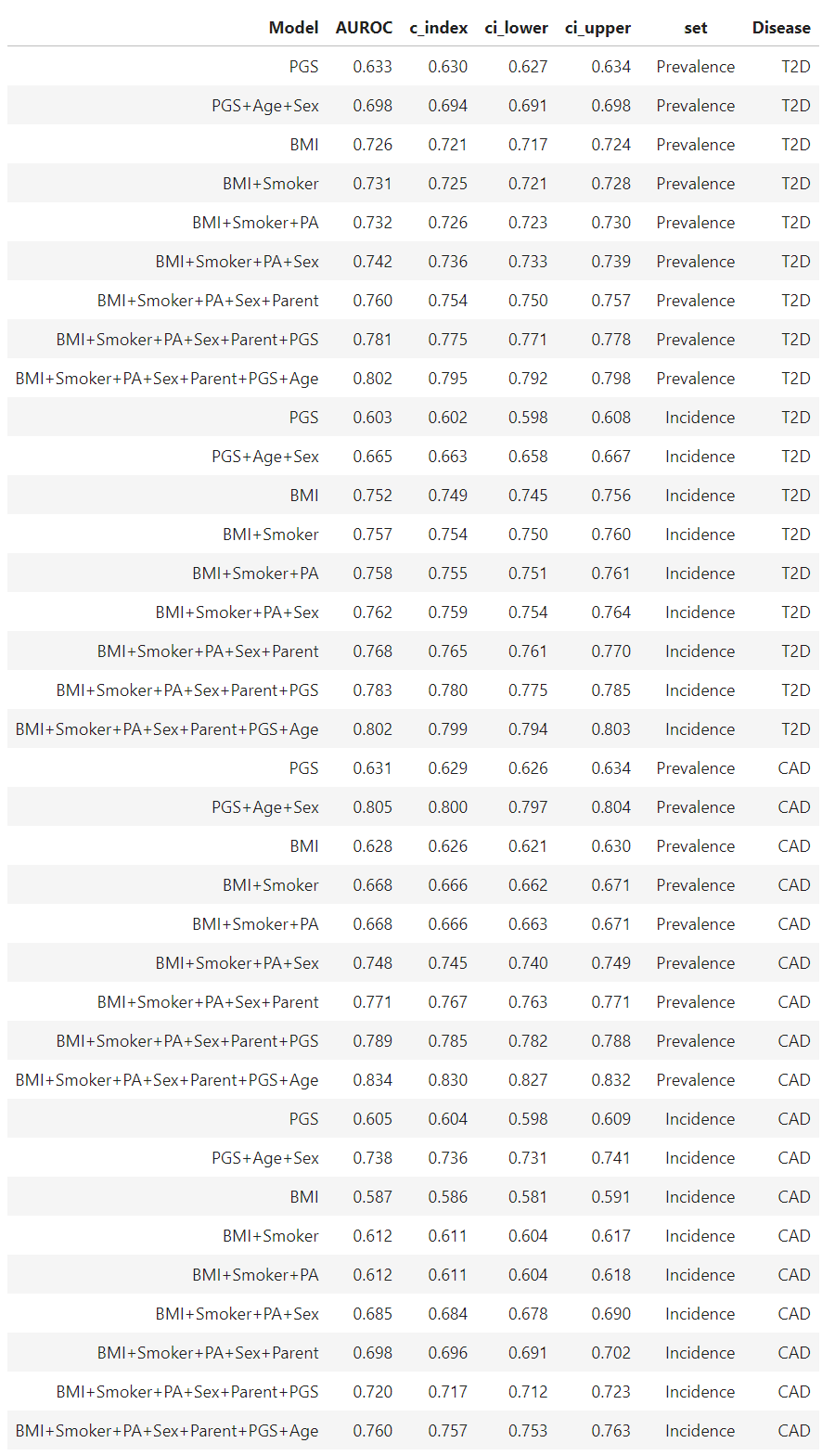


UKB odds ratios top decile:


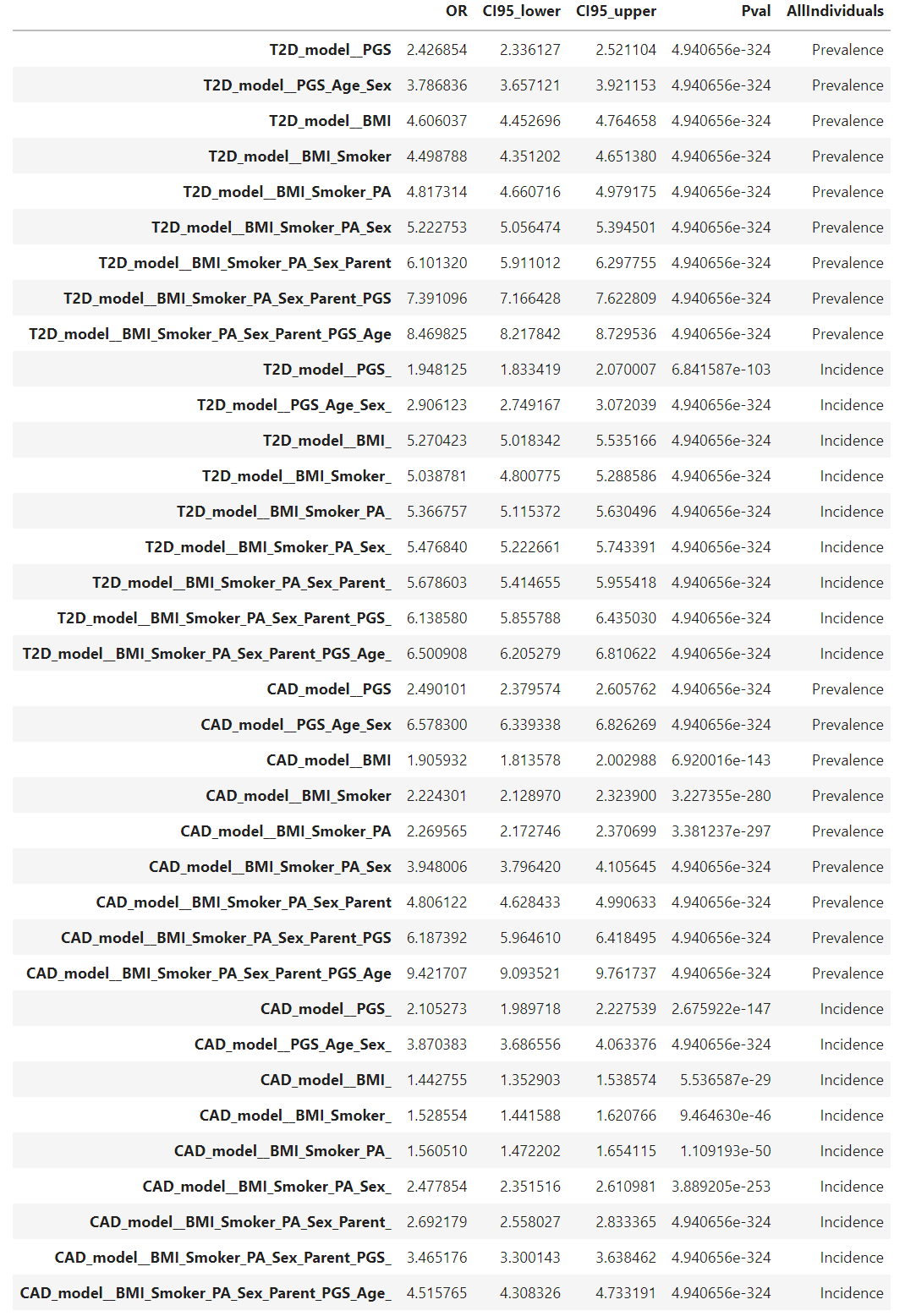


Supplementary table 2. Data-Fields used for calculating disease incidence and the associated age or date of diagnosis Data-Fields.

| Diagnosis | | Age or date of diagnosis | |
| --- | --- | --- | --- |
| Data-Field | Name | Data-Field | Name |
| 20002 | Non-cancer illness code, self-reported | 20009 | Interpolated Age of participant when non-cancer illness first diagnosed |
| 20004 | Operation code | 20011 | Interpolated Age of participant when operation took place |
| 2443 | Diabetes diagnosed by doctor | 2976 | Age diabetes diagnosed |
| 6150 | Vascular/heart problems diagnosed by doctor | 3894 | Age heart attack diagnosed |
| 41271 | Diagnoses – ICD9 | 41281 | Date of first in-patient diagnosis - ICD9 |
| 41270 | Diagnoses - ICD10 | 41280 | Date of first in-patient diagnosis - ICD10 |
| 41272 | Operative procedures - OPCS4 | 41282 | Date of first operative procedure - OPCS4 |

References

1. Liu, W., Zhuang, Z., Wang, W., Huang, T. & Liu, Z. An Improved Genome-Wide Polygenic Score Model for Predicting the Risk of Type 2 Diabetes. *Front. Genet.* **0**, 63 (2021).

2. Privé, F., Vilhjálmsson, B. J., Aschard, H. & Blum, M. G. B. Making the Most of Clumping and Thresholding for Polygenic Scores. *Am. J. Hum. Genet.* **105**, 1213–1221 (2019).

3. Shin Heng Mak, T. *et al.* Polygenic scores via penalized regression on summary statistics. (2017) doi:10.1002/gepi.22050.

4. Privé, F., Arbel, J. & Vilhjálmsson, B. J. LDpred2: better, faster, stronger. *Bioinformatics* **36**, 5424–5431 (2021).

5. Lloyd-Jones, L. R. *et al.* Improved polygenic prediction by Bayesian multiple regression on summary statistics. *Nat. Commun. 2019 101* **10**, 1–11 (2019).

6. Kulm, S., Marderstein, A., Mezey, J. & Elemento, O. A Systematic Framework for Assessing the Clinical Impact of Polygenic Risk Scores. *SSRN Electron. J.* (2021) doi:10.2139/SSRN.3808292.

7. Mostafavi, H. *et al.* Variable prediction accuracy of polygenic scores within an ancestry group. *Elife* **9**, (2020).

8. Khera, A. V. *et al.* Genome-wide polygenic scores for common diseases identify individuals with risk equivalent to monogenic mutations. *Nature Genetics* vol. 50 1219–1224 (2018).

9. Mars, N. J. *et al.* Polygenic and clinical risk scores and their impact on age at onset of cardiometabolic diseases and common cancers. *bioRxiv* 727057 (2019) doi:10.1101/727057.

10. McCarthy, S. *et al.* A reference panel of 64,976 haplotypes for genotype imputation. *Nat. Genet.* **48**, 1279–1283 (2016).

11. Duncan, L. *et al.* Analysis of polygenic risk score usage and performance in diverse human populations. *Nat. Commun.* **10**, 1–9 (2019).

12. Marnetto, D. *et al.* Ancestry deconvolution and partial polygenic score can improve susceptibility predictions in recently admixed individuals. *Nat. Commun. 2020 111* **11**, 1–9 (2020).

13. Mars, N. *et al.* Polygenic and clinical risk scores and their impact on age at onset and prediction of cardiometabolic diseases and common cancers. *Nat. Med.* **26**, 549–557 (2020).
